# Body composition changes over a 4-year span in American college football players

**DOI:** 10.3389/fnut.2026.1901586

**Published:** 2026-07-30

**Authors:** Tamara Hew-Butler, Keegan Pulford-Thorpe, Valerie Smith-Hale, Ruben Mendoza, Jordan Sabourin

**Affiliations:** 1 Warrior Performance Laboratory, Exercise and Sport Science, Wayne State University, Detroit, MI, United States; 2 Nutrition and Food Science Department, Wayne State University, Detroit, MI, United States; 3 Athletics Department, Wayne State University, Detroit, MI, United States; 4 Sports Performance, Detroit Pistons Organization, Detroit, MI, United States

**Keywords:** American football, DXA scan, linemen, resistance training, sports performance

## Abstract

American collegiate football players (13 lineman; 14 non-lineman) underwent total body composition assessment using dual energy x-ray absorptiometry during August football camp in 2019 (Year 1/baseline); 2021 (Year 3); and 2022 (Year 4). Data were not collected in 2020 (Year 2) due to COVID. Main outcome measures included: total mass (TM); lean mass (LM); fat mass (FM); and bone mass, assessed as bone mineral density/BMD). Lineman had significantly more TM (124 ± 15 vs. 89 ± 15 kg; *p* < 0.001), LM (90 ± 6 vs.72 ± 10 kg; *p* < 0.001), and FM (31 ± 9 vs. 15 ± 5 kg; *p* < 0.001) versus non-lineman. 2-way ANOVA demonstrated no difference between Groups (linemen vs. non-linemen) but significant (*p* < 0.001) increases in LM (3.3 kg ± 2.4/4.1%) and BMD (0.04 ± 0.04 g/cm/3%) across Time. Correlations between LM vs. TM were significant (*p* < 0.0001) for linemen (*r*^2^ = 0.80) and non-linemen (*r*^2^ = 0.98), with the regression slopes intersecting at a TM “breakpoint” of 104 kg. A positive correlation was noted between the 4-year change (Δ) in LM Δ vs. FM Δ for linemen only (*r*^2^ = 0.54; *p* = 0.004), with slope equations predicting a 1.8 kg increase in FM for every 1 kg increase in LM. In conclusion, longitudinal body composition gains are modest (3–4%) with linemen gaining more FM (2:1) than LM, above a 104 kg TM threshold. Our modest sample size requires confirmation in larger cohorts.

## Highlights

- Realistic annual weight gain targets (for TM, LM, and BM) should not exceed 1–2% without the risk of excessive fat accumulation, in American football players undergoing supervised team training.
- Very large (>104 kg), resistant-trained, American football players (i.e., linemen) appear to gain twice as much fat (2 kg) to accompany 1 kg of LM tissue accrual (2 kg FM:1 kg LM), in response to long-term (>1 year) resistance training.
- An estimated energy surplus of 200 kcal/day is hypothetically required to elicit a 1 kg increase in LM, across a 3-month cycle of resistance training, in large (>104 kg) football players.

## Introduction

American football (referred to as “football” in this paper) is the highest revenue-generating sport at the collegiate level (1). Given the vast individual (2) and program (1) revenue potential associated with winning college football programs, the primary off-season training and nutritional goals are to cultivate bigger—and thereby stronger—football players (3–5). A link between size and strength is supported by a study conducted on 53 collegiate football players, whereas a 10.1 kg increase in total body mass (i.e., bodyweight) resulted in a 10% increase in power (6). As such, linemen at one collegiate football program are expected to increase bodyweight by 20–30% in their first 2 years on campus to improve performance (3). Expectations for players (and performance coaches) to continuously increase body mass over time (3, 7) thereby likely carries health, performance, and heightened financial risks.

National Collegiate Athletic Association (NCAA) football players participate in organized team training throughout the 9-month school year, with off-season training (i.e., January–May) geared toward maximizing size and strength (3, 7–9). Longitudinal studies demonstrate a “ceiling effect” whereas significant increases in body size appear to plateau after the first year of intensive resistance training (8). Muscle biopsy studies corroborate with longitudinal body composition findings whereas muscle fiber hypertrophy appears constrained after 3–6 months of resistance training in highly-trained bodybuilders, power lifters, and weightlifters (10–13). Conversely, another longitudinal study, following 13 NCAA Division I (DI) football players across a 4-year playing career demonstrated modest bodyweight gains (6.6 kg) after 4-years of organized team training (9).

Body composition analyses, in addition to body mass measurement using dual energy x-ray absorptiometry (DXA) scans, identified the presence of a “breakpoint” whereas athletes gain disproportionately more fat than lean mass in attempt to gain bodyweight (3, 14). One cross-sectional study, performed on 53 DI football players, identified 104 kg (229 lbs) as the total body mass “breakpoint” (3). A similar study performed on 370 professional national football league (NFL) players identified a higher body mass breakpoint of 114 kg (251 lbs) whereas greater fat mass (than lean mass) contributed to further body mass gains (14). The downside of any excess fat accumulation (above this metabolic “breakpoint”), however, includes increased risk for metabolic syndrome (15), heatstroke (16), and/or injury (17) to large football players (e.g., linemen).

Thus, the primary aims of this study are to (1) assess longitudinal changes in body composition over a 4-year span (typical collegiate career), (2) investigate if larger players (linemen) accumulate a greater proportion of fat than smaller players (non-linemen) over time and training, and (3) estimate the ratio of fat to lean mass accrual over 4-years in a cohort of NCAA Division II (DII) football players.

## Methods

This longitudinal study was part of an ongoing cross-sectional and longitudinal screening project approved by the University’s institutional Review Board (IRB#073919M1E). Inclusion criteria included all collegiate American football players competing at a single NCAA DII midwestern University. Exclusion criteria included all persons who were not an official member of the University’s football team at the time of testing. All participating football players (*N* = 27) represented DII players, from a midwestern university, who signed voluntary written informed consent prior to participation in 2019 (year one/1), 2021 (year three/3), and 2022 (year four/4) DXA scans for annual total body composition analyses. Of note, lab testing was suspended in 2020 (year two/2) due to COVID-19 restrictions on all in-person training and competition. Thus, only three serial DXA scans were conducted over a 4-year span (i.e., a typical collegiate career) and were completed in August, during official football training camp.

After signing written informed consent, height and weight were measured using a stadiometer with a weight beam scale (Detecto, Webb City, MO). Total body mass (TM), lean mass (LM), fat mass (FM), and bone mass (BM; reported as bone mineral density/BMD) were measured using a whole-body DXA (Horizon A, Hologic™, Bedford, MA; software version 13.6.0.5; TBAR1209 calibration). All whole body DXA scans and analyses were performed by the same investigator, with participants wearing only compression shorts. All jewelry was removed, and the compression shorts were free of any metal or metallic logos which would artificially contribute to bone mass. Neither food, fluid intake, nor prior exercise could be controlled, prior to each scan, as the recommended (18).

Each participant was positioned on the DXA scan table supine, with the arms placed adjacent to the torso, palms perpendicular to the scan table, with the feet positioned as close together as possible (and secured with tape) to accommodate the larger size of our participants. This compact positioning allowed us to focus on total mass results, rather than regional results. For participants taller than 1.96 m (exceeding scan table height dimensions), the head was included within the confines of the DXA scan area, with portions of the feet excluded to maximize total mass comparisons (19). One participant (146 kg), at one timepoint (2019), exceeded the width (1.1 m) of the scan table. For this one player, one-time (1.2% of all data analyzed), a “reflection” software adjustment was utilized to estimate overall total body mass and body composition (LM, FM, and BM) parameters, by “mirroring” the right side of the body to calculate the left side (20).

The percent coefficient of variation (CV%) of the Horizon A device was calculated (in-house) as: total mass = 0.10%; FM = 0.46%; LM = 0.49%; bone mineral content (BMC) = 0.86%; and body fat % = 0.43%.

*Statistical analyses*: Body mass (TM/bodyweight) and total body composition (LM, FM, and BMD) variables passed (*p* > 0.05) the assumptions of normality, using the Shapiro-Wilks test for the total cohort (*N* = 27). The cohort was then subdivided into two groups for analyses: the linemen group (*n* = 13; consisting of offensive and defensive linemen) and the non-linemen group (*n* = 14; consisting of all position players other than linemen). Unpaired *t*-tests were used to compare linemen versus (vs.) non-linemen for cross-sectional demographic and body composition variables.

A 2-way ANOVA was utilized to compare changes (∆) in body composition variables over both Time (training year) and between Groups (linemen vs. non-linemen). The change (∆) values represent differences between the third year minus year 1 (2021–2019 values) to represent the “year 3” ∆-value and the difference between year 4 minus year 1 (2022–2019 values) to represent the “year 4” ∆-value. No data were collected in year 2 (2020) due to COVID-19 related restrictions.

Correlation analyses were utilized to assess relationships between body composition and body mass variables, both at one point in time (cross-sectional data obtained in year 4) and for the ∆ values calculated over a 4-year span (2022–2019). The cross-sectional correlations (from 2022; year 4) were used to identify a “breakpoint” in total body mass for both FM and LM). This purported inflection point represents the intersection point of the linear regression lines for the linemen and non-linemen cohorts, as previously estimated by Bosch (14). The slope equations for the longitudinal ∆ data correlations were then utilized to estimate the ratio of FM to LM gains, over a 4-year span (*y* = *mx* + *b*; whereas *y* = lean mass ∆, *X* = fat mass ∆, and *b* = *y*-intercept). We report the coefficient of determination (*r*^2^) to estimate the proportion of variation the independent variable has on the dependent variable, in all linear correlation analyses.

**Figure 1:**
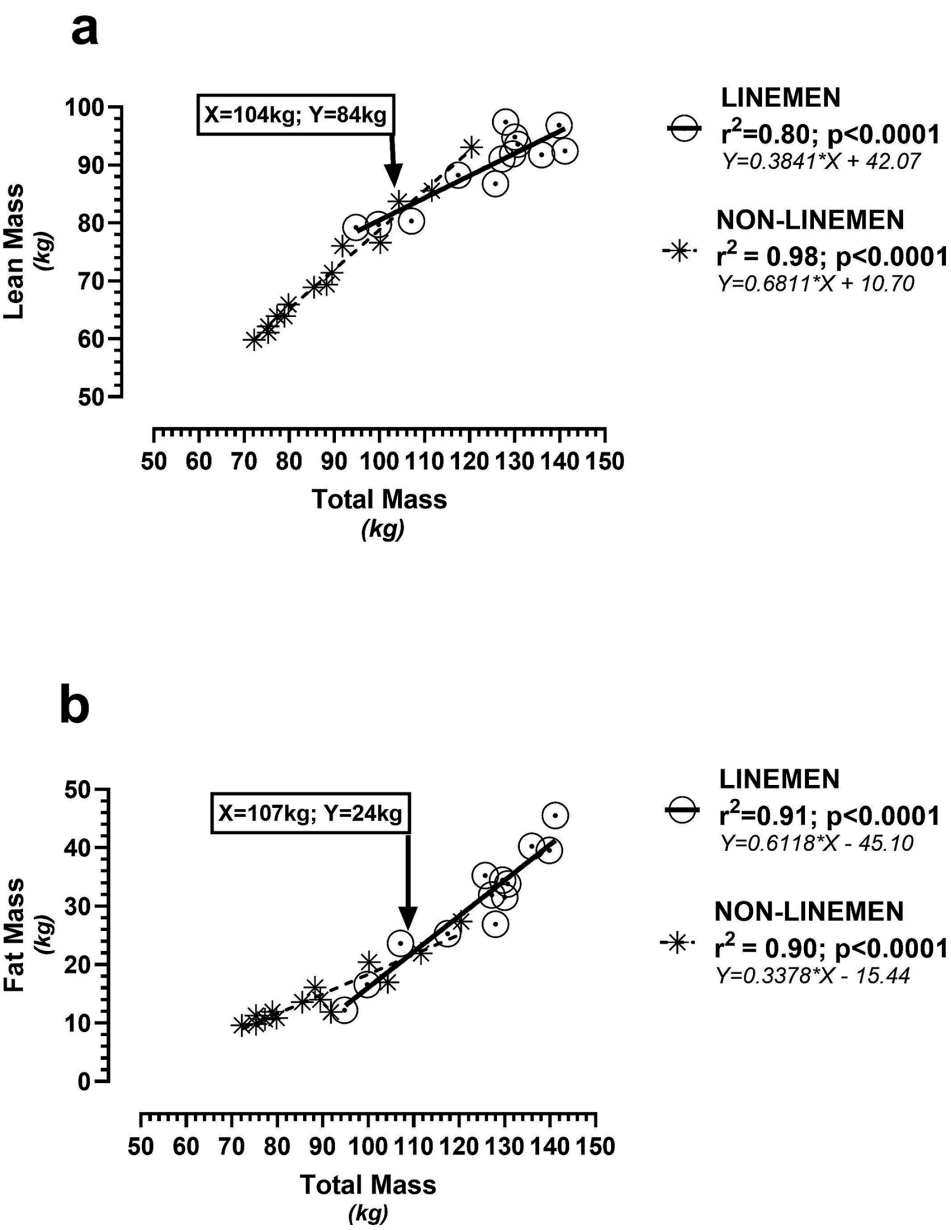
Relationships between total lean mass versus total body mass **(a)** and total fat mass versus total body mass **(b)** separated into linemen (*n* = 13) and non-linemen (*n* = 14) player groups.

All data presented as mean ± standard deviation (SD) with statistical significance set *a priori* at *p* < 0.05. We report 95% Confidence intervals (CI) for the slopes and intersection points for the linemen versus non-linemen linear regression lines (Figures 1a,b) to highlight the potential variance surrounding these mathematical “breakpoint” estimations. A power analyses was not conducted, as the limitations on sample size were beyond our control (i.e., how many players remained on the team, over 4-years) but was double the number of participants used in a similar longitudinal analysis, which followed 13 total players (9).

## Results

The mean values for demographic and body composition variables obtained in year 4 (2022) are reported in Table 1, for the total cohort (*N* = 27) and then subdivided into the linemen (*n* = 13) and non-linemen (*n* = 14) player groups. The linemen players were significantly (*p* < 0.05) larger, with greater amounts of LM and FM, compared with non-linemen players.

**Table 1:** Demographic and body composition data on 27 total (combined) football players, subdivided into linemen versus non-linemen position groups.

Variable	Linemen (*n* = 13)	Non-linemen (*n* = 14)	Total (*N* = 27) (min-max)
Age (years)	21.6 ± 0.9	21.6 ± 0.9	21.6 ± 0.9 (20–23)
Height (m)	1.89 ± 0.038^**^	1.83 ± 0.06	1.86 0.06 (1.72–1.94)
Total mass (kg)	123.6 ± 14.7^***^	89.3 ± 14.8	105.9 ± 22.7 (72–141)
Lean mass (kg)	89.6 ± 6.3^***^	71.5 ± 10.2	80.2 ± 12.4 (60–97)
Fat mass (kg)	30.5 ± 9.4^***^	14.7 ± 5.3	22.3 ± 10.9 (10–46)
Body fat (%)	24.2 ± 5.3^***^	16.1 ± 3.0	20.0 ± 5.9 (13–32)
BMD (g/cm^2^)	1.38 ± 0.12	1.35 ± 0.11	1.36 ± 0.11 (1.18–1.62)
Z-score	1.37 ± 0.81	0.87 ± 0.96	1.12 ± 0.91 (−0.7–3.2)

^*^p < 0.05; ^**^p < 0.01; ^***^p < 0.001 between linemen vs. non-linemen.

These data were collected in 2022 (final data collection, just prior to their fourth year playing collegiate football). All data reported as means ± standard deviation (SD), with statistical significance between linemen versus non-linemen noted as asterisks.

**Figure 2:**
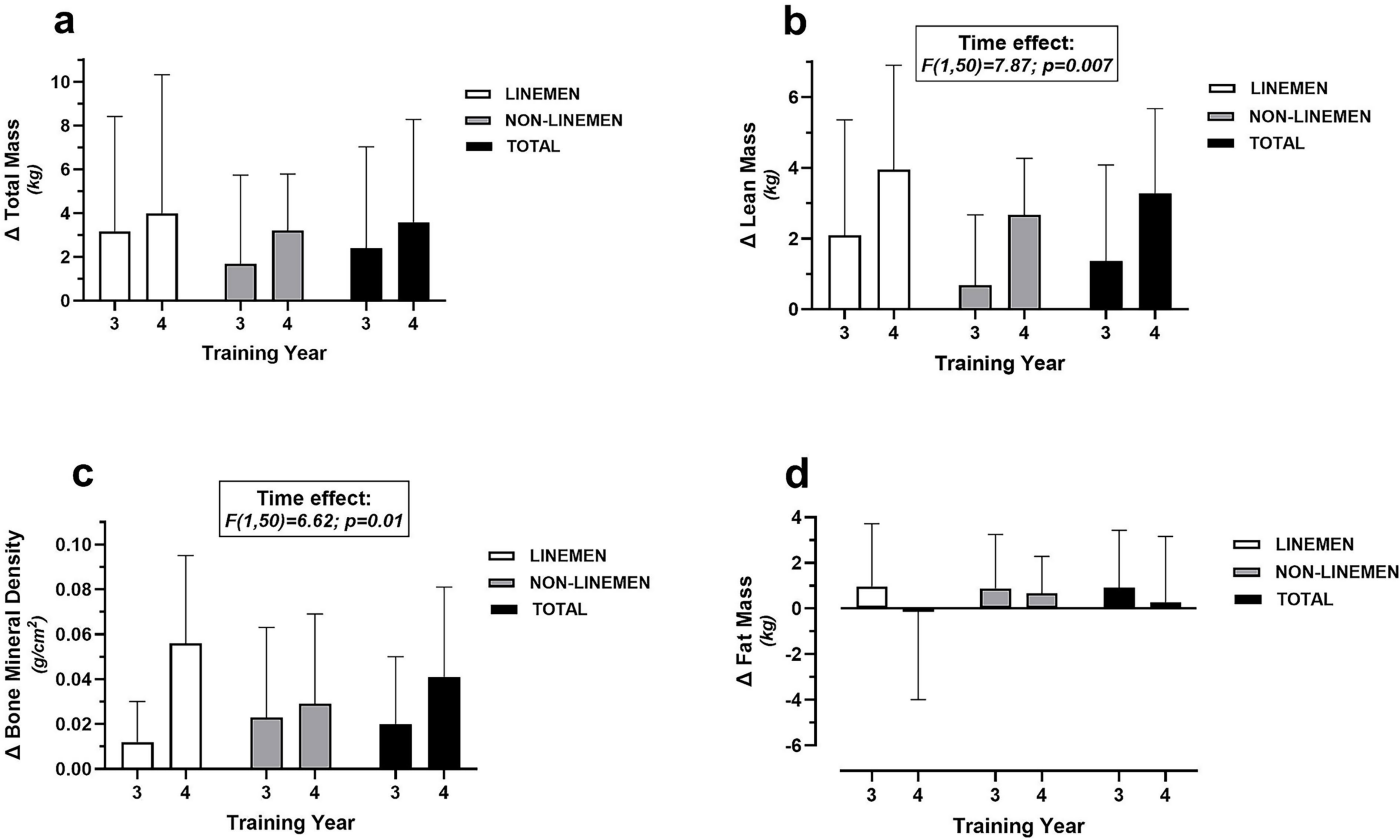
Longitudinal changes (∆) in total body mass **(a)**, total lean mass **(b)**, bone mineral density (BMD) **(c)**, and total fat mass **(d)** in 27 football players (TOTAL), further subdivided into linemen (*n* = 13), and non-linemen (*n* = 14) position groups. The change (∆) values represent differences from the start of August training camp in year three (2021) and training year four (2022) minus the baseline levels (2019). All data reported as means ± standard deviation (SD). Training and competition were suspended in 2020 by COVID-19; thus, no data were collected in year two.

Figure 2 details the changes in TM (a), LM (b), BMD (c), and FM (d) across both Group (linemen vs. non-linemen) and Time (∆’s at year 3 and year 4). Significant Time effects were noted for both LM ∆ and BMD ∆ whereas significant increases in LM and BMD were noted between years 3 and 4, for both linemen and non-linemen. No significant (*p* > 0.05) Group (linemen vs. non-linemen) nor Interaction (i.e., Group × Time) effects were noted for any body mass or body composition variable, according to the 2-way ANOVA results.

Figure 1 depicts the linear correlations for both lineman and non-linemen players for LM vs. TM (a) and for FM vs. TM (b). The correlations for both linemen and non-linemen players were highly significant (*p* < 0.0001). For the LM vs. TM correlation (Figure 1a), the slope for the linemen players was 0.38 (0.26–0.51; 95% CI) while the slope for the non-linemen players was 0.68 (0.62–0.74). For the FM vs. TM correlation (Figure 1b), the slope for the linemen players was 0.61 (0.48–0.74) while the slope for the non-linemen players was 0.34 (0.27–0.41). Plainly speaking, from these slope analyses, the linemen players gained more FM and less LM, above a slope intersection point with TM. In our cohort, the intersection of the regression lines occurred between 104 kg (96–112 kg; 95% CI for LM; Figure 1a) and 107 kg (99–115 kg CI for FM; Figure 1b).

**Figure 3:**
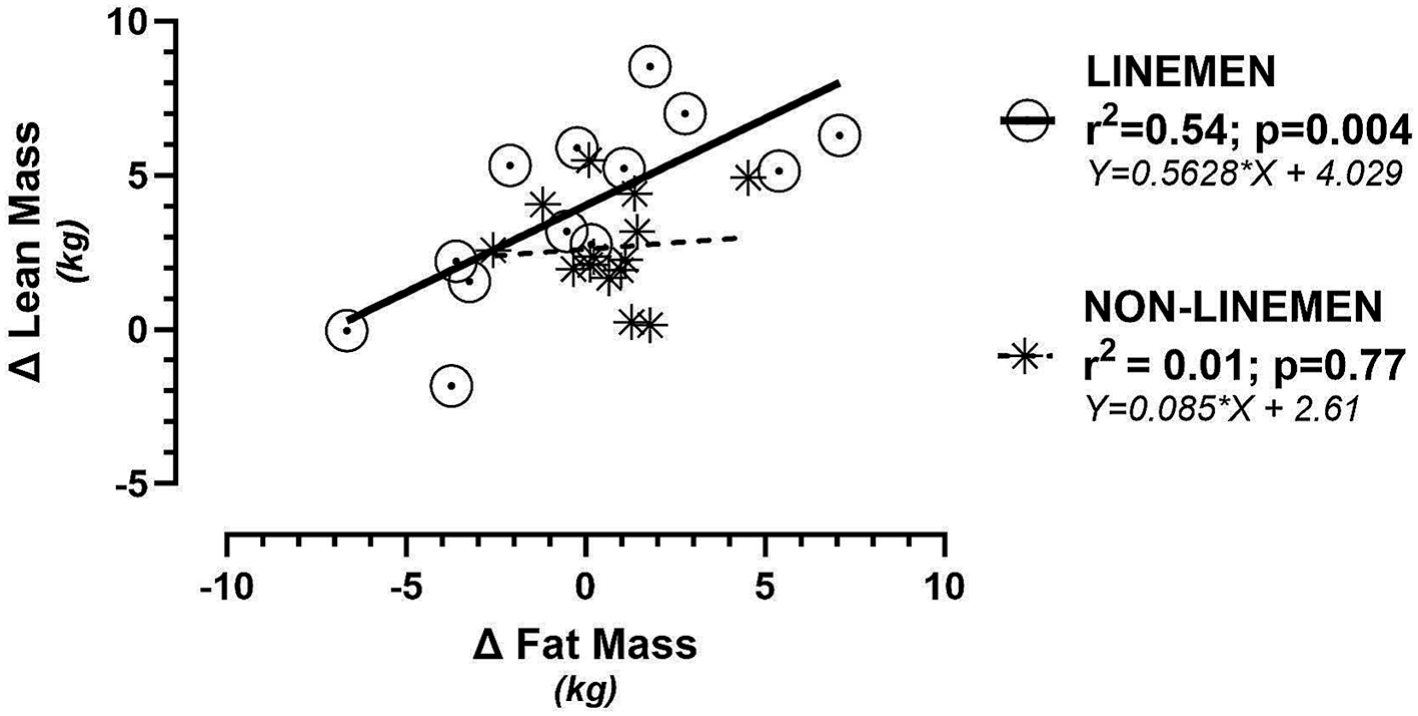
Relationship between the change (∆) in total lean mass versus the ∆ in total fat mass across 4-years of study (2022 minus 2019 values), for 27 football players separated into linemen (*n* = 13) and non-linemen (*n* = 14) player groups.

Figure 3 demonstrates a significant positive linear relationship between the LM ∆ vs. FM ∆, across 4-years, for the linemen player group only. For the linemen group, 54% of the variance seen in LM changes were associated with changes in FM. Using the slope equation for the linear relationship for the linemen players only (*n* = 13), for every 1 kg increase in LM was a proportional 1.8 kg increase in FM (above a 4.0 kg lean mass increase, as noted by the y-intercept). For the non-linemen player group, no linear relationship (0.0% variance) was noted between LM ∆ vs. FM ∆ (thus, no FM:LM ratio estimations were calculated).

## Discussion

Our longitudinal data suggests DII football players gain an average of 3.6 kg (3.3% overall increase from year 1) of TM over a 4-year span. Most of the TM increases were driven by significant increases in LM (3.3 kg; or 4.1% increase across 4-years) and BMD increases (0.04 g/cm^2^ or 3% increase over 4-years) for both lineman and non-linemen players. The 4-year increases in TM, LM, and BMD were smaller in magnitude than previously reported in a cohort of 13 DI players which demonstrated a 6.6 kg increase in TM, 4.3 kg increase in LM, and 0.03 g/cm^2^ increase in BMD over a similar timeframe (4-years) (9). We hypothesize that DI teams may have access to greater nutritional resource allocation–such as dedicated dietetic support staff—which may have contributed to the attenuated gains seen in our DII players (21). Furthermore, football players competing at the DI level are often larger, faster, and more skilled than DII players due to variations in training volume, recruitment quality, and genetic endowments that may promote greater lean mass and tissue accretion. Nevertheless, our data do support the findings of Trexler et al. whereas modest (3–4%) gains in lean and bone mass are, indeed, expected in football players (regardless of NCAA Division level) over a 4-year collegiate trajectory, that are favorable to health (osteogenesis) (22) and performance (increased strength) (5, 9).

Our football players gained a greater amount of LM during the second two-years (1.9 kg) than over the first two years (1.4 kg). Results from past studies conflict with the current study findings, with previous studies (performed on DI football players) reporting the largest gains in both size and strength within the initial 1–2 years of training (5, 8, 23). We believe that our conflicting findings result from the abrupt restrictions on training and competition in year 2 due to COVID-19. Alternatively, Hoffman J et al. reported a biphasic response in size and strength (particularly in linemen) within DIII football players whereas the biggest size gains were found during the first and fourth years of training (24). These alternative findings suggest that secondary factors (i.e., genetics, nutrition, prior training, habitual physical activity) may exacerbate variabilities in lower (DII and DIII) versus higher (DI) players (genetics) and programs (environment) (25, 26).

Given the modest average increases in LM reasonably expected over a 4-year collegiate trajectory (9), more substantial (>5%) bodyweight gains must logically accrue as fat. As such, a study performed on 370 NFL players demonstrates that beyond a “breakpoint” bodyweight of 114 kg (~250l bs), players accrue exponentially more FM within the subcutaneous and visceral fat compartments than players weighing less than 114 kg (14). Similarly, a study performed on 53 DI players defined a threshold “breakpoint” bodyweight of ~104 kg (3). Our theoretical TM “breakpoint” of 104-107 kg (Figures 1a,b) was remarkably similar to the threshold proposed in Binkley et al. (3). We believe that the closeness in age (22 vs. 20 years; our results vs. Binkley’s results, respectively) and similarities in bodyweight of collegiate linemen cohorts (124 kg vs. 122 kg) attributed to the similar “breakpoint” estimations. The higher “breakpoint” TM threshold of 114 kg for NFL players was likely attributed to the older age (24 years) and TM (137 kg) of professional football players (14).

To further extend our “breakpoint” analyses, we used our linear regression model to estimate the ratio of expected FM to LM gains over a 4-year timespan. We calculated that our linemen football players gain ~1.8 kg of FM in proportion to every 1 kg increase in LM (Figure 3). This roughly 2:1 kg (FM:LM) total mass accrual ratio is similar to findings derived from a 100-day overfeeding study, performed on 12 pairs of identical twins (27). Although this was not a training study, the overfeeding intervention resulted in a 5.4 kg of fat mass gain concomitant with a 2.7 kg fat-free mass gain (or 2:1, FM:LM accrual) (27). These collective results indirectly support the need for an energy surplus to meet the high metabolic requirements of increasing muscularity, as suggested previously (28, 29). Of note, it is possible for underfit and overfat individuals to gain muscle mass while losing body fat (“body recomposition”) through training and dietary modifications (30). However, the body mass (and composition) thresholds for which the energetic requirements would switch from surplus (lose fat with LM gains) to deficit (gain FM to support LM gains) requires more critical, tissue-specific and metabolic, inquiry.

The physiological and nutritional mechanisms explaining why our largest (>104 kg) football athletes gain almost twice as much FM than LM, in attempt to “gain weight” through resistance exercise, may be explained by evidence supporting the existence of a genetically determined plateau in muscle cell growth (31). Evidence suggests that muscle cells can regulate their own size (scaling) and initiate molecular braking systems when myocytes achieve a (pre-programmed) maximal size (31). It remains unclear if limitations on muscle cell hypertrophy result from anabolic resistance (i.e., elevated resting myostatin levels), an increase in catabolic (proteolytic) processes, or both (31). The contributions of nutritional strategies and energetic needs—which may augment or attenuate muscle growth—requires further investigation.

Hypothetically, from a caloric perspective, if the energy content of fat mass is 9,300 kcals/kg (and 1,020 kcals/kg for lean mass) (27), then a linemen player would require an energy surplus of 17,760 kcals to gain 1 kg of LM (i.e., 9,300 kcals of FM x 1.8 + 1,020 kcals of LM) during training. Extrapolating further, for a typical 3-month (90-day) training block, linemen athletes would then require an extra caloric intake of 197 kcals/day to support the accrual of 1 kg of LM during targeted resistance training. This estimation is below the recommended energy surplus caloric estimations of 359–478 kcal/day (1,500–2,000 kJ/day), to increase LM accrual, as proposed by Slater et al. (28). However, given that the resting metabolic rate (RMR) for linemen players is around 2,841 kcal/day (32) coupled with the average energy expenditure (for offensive linemen) during practice is 1,677 kcal/day (33), the addition of ~200 kcals on top of estimated daily caloric needs of 5,000–6,000 kcals/day is a daunting task for both athletes and sports dietitians for our largest (>104 kg) athletes. Attainment of a daily energy surplus, in athletes trying to gain LM, is not trivial as a 2021 meta-analysis suggested that an energy deficit of 500 kcal/day will prohibit gains in LM from resistance training (34). Thus, adequate nutrient intake is an important consideration to promote and support tissue accretion. However, without dietary control, we cannot assert that the observed changes seen in our cohort were caused by training, dietary factors (including caloric surplus), or (likely) both.

There are several limitations of this study. First, it is unknown how the prolonged COVID-19 disruptions on training, dietary habits, competition, and data collection (year 2) may have altered both the trajectory and magnitude of body mass and composition gains over time. As such, the forced detraining in 2020 may have distorted the “natural” adaptation trajectory, so that in 2021 we were seeing a partial recovery of lost conditioning rather than planned growth. However, it has been shown that muscle size (and strength—not measured here) can rebound quickly (~6-weeks) after periods of detraining, (35, 36). Additionally, another study conducted on 29 NCAA D1 American football players concluded that COVID-19 restrictions on training had no detrimental effects on body composition (37). Thus, the impact of COVID-19 disruptions on training, dietary choices, and body composition remain unclear. Second, corresponding strength metrics (i.e., back squat, bench press, and power clean) were not collected as these variables were not included within the IRB-approved protocol. Third, we were not able to standardize (or control) food or fluid intake, nor exercise, prior to each DXA scan. We performed DXA scans when there was a suitable timeframe approved by the head football coach, as to not interfere with any scheduled August football training camp activities. Fourth, we did not collect nutritional, supplement, or potential doping information which would have illuminated critical information on both macronutrient and caloric intake on body composition changes over time (38). Finally, our modest sample size (*N* = 27), coupled with the high inter- and intra-variability between different positional players, limits the strength of our overall conclusions to wider cohort of collegiate football athletes and requires confirmation in larger cohorts. Furthermore, the coefficient of determination *r*^2^ = 0.54 for the FM:LM relationship (Figure 3) explained only half of the variance which limits the strength of our interpretation. However, our cohort was larger than a previous study sample (*N* = 13) and underscores the current (and future) difficulty of finding elite college athletes who commit to a single program over a 4-year playing career. We also did not include a control group of age-matched, non-athlete, young adult males to disentangle training-induced and dietary adaptations from normal developmental changes expected in this age range. Future investigations should provide details on programmatic training periodization as well as individual disruptions to training (i.e., injury and illness); all of which likely impacted body composition changes over 1–4 years of training.

In conclusion, both linemen and non-linemen football players gain ~3% of TM, ~4% of LM, ~3% of BM, and ~1% of FM over a 4-year span of intensive training to maximize size and strength over time. Although the increases in LM and BMD were statistically significant the average gains were modest (3–4%, or ~1% per year) and below expectations for many college programs seeking bigger, better, players (3, 7). Additionally, for linemen weighing above 104 kg (230 lbs), a greater proportion of bodyweight accrues as FM, in an estimated 2:1 proportion to LM (i.e., 2 kg of fat accompanies every 1 kg increase in lean mass). Thus, expectations for the average incoming collegiate football player to gain over 4% of TM, LM or BMD across a 1–2-year period in response to high quality strength training appears unrealistic without higher proportions of body mass gained as fat. The impacts of dietary intake and caloric expenditure on these longitudinal changes in body composition remain unclear, and warrants further study.

## Data Availability

The raw data supporting the conclusions of this article will be made available by the authors, without undue reservation.

## References

[1] Hughes G. (2024). College athletics' 25 powerhouses who produce the most revenue entering 2024. 247SPORTS. Available online at: https://247sports.com/longformarticle/college-athletics-25-powerhouses-who-produce-the-most-revenue-entering-2024-224490000/ (Accessed July 8, 2026).

[2] Network CS. (2024). Top 10 NIL Deals in 2024: Shedeur Sanders, Travis Hunter Making Prime Time Proud. Available online at: https://collegefootballnetwork.com/top-10-nil-deals-in-2024/ (Accessed July 8, 2026).

[3] Binkley TL Daughters SW Weidauer LA Vukovich MD. Changes in body composition in division I football players over a competitive season and recovery in off-season. J Strength Cond Res. (2015) 29:2503–12. doi: 10.1519/JSC.0000000000000886, 26313574

[4] Secora CA Latin RW Berg KE Noble JM. Comparison of physical and performance characteristics of NCAA division I football players: 1987 and 2000. J Strength Cond Res. (1987) 18:286–91. doi: 10.1519/R-12852.1, 15142002

[5] Miller TA White ED Kinley KA Congleton JJ Clark MJ. The effects of training history, player position, and body composition on exercise performance in collegiate football players. J Strength Cond Res. (2002) 16:44–9. doi: 10.1519/1533-4287(2002)016<0044:TEOTHP>2.0.CO;2 11834106

[6] Mayhew LJ Piper FC Schwegler TM Ball TE. Contributions of speed, agility and body composition to anaerobic power measurement in college football players. J Appl Sport Sci Res. (1989) 3:101–6.

[7] Wichmann TK Wolfson J Roelofs EJ Bosch TA Bach CW Oliver JM . Longitudinal assessment of NCAA division I football body composition by season and player age. J Strength Cond Res. (2022) 36:1682–90. doi: 10.1519/JSC.0000000000004256, 35333212

[8] Stodden DF Galitski HM. Longitudinal effects of a collegiate strength and conditioning program in American football. J Strength Cond Res. (2010) 24:2300–8. doi: 10.1519/JSC.0b013e3181dc4255, 20703166

[9] Trexler ET Smith-Ryan AE Mann JB Ivey PA Hirsch KR Mock MG. Longitudinal body composition changes in NCAA division I college football players. J Strength Cond Res. (2017) 31:1–8. doi: 10.1519/JSC.0000000000001486, 28005635 PMC4878695

[10] MacDougall JD Sale DG Elder GC Sutton JR. Muscle ultrastructural characteristics of elite powerlifters and bodybuilders. Eur J Appl Physiol Occup Physiol. (1982) 48:117–26. doi: 10.1007/BF00421171, 7199447

[11] MacDougall JD Elder GC Sale DG Moroz JR Sutton JR. Effects of strength training and immobilization on human muscle fibres. Eur J Appl Physiol Occup Physiol. (1980) 43:25–34. doi: 10.1007/BF00421352, 7371625

[12] Alway SE Grumbt WH Stray-Gundersen J Gonyea WJ. Effects of resistance training on elbow flexors of highly competitive bodybuilders. J Appl Physiol (1985). (1992) 72:1512–21. 1592744 10.1152/jappl.1992.72.4.1512

[13] Häkkinen K Pakarinen A Alen M Kauhanen H Komi PV. Neuromuscular and hormonal adaptations in athletes to strength training in two years. J Appl Physiol (1985). (1988) 65:2406–12. doi: 10.1152/jappl.1988.65.6.2406, 3215840

[14] Bosch TA Burruss TP Weir NL Fielding KA Engel BE Weston TD . Abdominal body composition differences in NFL football players. J Strength Cond Res. (2014) 28:3313–9. doi: 10.1519/JSC.0000000000000650, 25187247

[15] Steffes GD Megura AE Adams J Claytor RP Ward RM Horn TS . Prevalence of metabolic syndrome risk factors in high school and NCAA division I football players. J Strength Cond Res. (2013) 27:1749–57. doi: 10.1519/JSC.0b013e31827367cd, 22996023

[16] Anderson SA Eichner ER Bennett S Boden BP Colgate B Courson R . Preventing exertional heat stroke in football: time for a paradigm shift. Sports Health. (2025) 17:484–90. doi: 10.1177/19417381241260045, 38874455 PMC11569638

[17] McCaskie CJ Sim M Newton RU Heasman J Rogalski B Hart NH. Preseason body composition is associated with in-season player availability in elite male Australian footballers. J Strength Cond Res. (2023) 37:1089–95. doi: 10.1519/JSC.0000000000004368, 36730574

[18] Nana A Slater GJ Hopkins WG Halson SL Martin DT West NP . Importance of standardized DXA protocol for assessing physique changes in athletes. Int J Sport Nutr Exerc Metab. (2014) 26:259–67. doi: 10.1123/ijsnem.2013-0111, 24458265

[19] Hew-Butler T Jurczyszyn H Sabourin J VanSumeren M Smith-Hale V. Too tall for the DXA scan? Contributions of the feet and head to overall body composition. J Clin Densitom. (2022) 25:384–91. doi: 10.1016/j.jocd.2021.11.008, 34969607

[20] Center for Disease Control (CDC) and Prevention. Body Composition Procedures Manual 2021. (2021). ATLANTA, GA:NHANES p. 1–120.

[21] Slutzky N. (2018). Understand the Different College Football Divisions. Verified Athletics. Available online at: https://www.verifiedathletics.com/athlete-resources/understand-the-different-college-football-divisions/ (Accessed July 8, 2026).

[22] Almstedt HC Canepa JA Ramirez DA Shoepe TC. Changes in bone mineral density in response to 24 weeks of resistance training in college-age men and women. J Strength Cond Res. (2011) 25:1098–103. doi: 10.1519/JSC.0b013e3181d09e9d, 20647940

[23] Jacobson BH Conchola EG Glass RG Thompson BJ. Longitudinal morphological and performance profiles for American, NCAA division I football players. J Strength Cond Res. (2013) 27:2347–54. doi: 10.1519/JSC.0b013e31827fcc7d, 23238095

[24] Hoffman JR Ratamess NA Kang J. Performance changes during a college playing career in NCAA division III football athletes. J Strength Cond Res. (2011) 25:2351–7. doi: 10.1519/JSC.0b013e31821743df, 21747290

[25] Roberts MD McCarthy JJ Hornberger TA Phillips SM Mackey AL Nader GA . Mechanisms of mechanical overload-induced skeletal muscle hypertrophy: current understanding and future directions. Physiol Rev. (2023) 103:2679–757. doi: 10.1152/physrev.00039.2022, 37382939 PMC10625844

[26] Damas F Phillips S Vechin FC Ugrinowitsch C. A review of resistance training-induced changes in skeletal muscle protein synthesis and their contribution to hypertrophy. Sports Med. (2015) 45:801–7. doi: 10.1007/s40279-015-0320-0, 25739559

[27] Bouchard C Tremblay A Després JP Nadeau A Lupien PJ Thériault G . The response to long-term overfeeding in identical twins. N Engl J Med. (1990) 322:1477–82. doi: 10.1056/NEJM199005243222101, 2336074

[28] Slater GJ Dieter BP Marsh DJ Helms ER Shaw G Iraki J. Is an energy surplus required to maximize skeletal muscle hypertrophy associated with resistance training. Front Nutr. (2019) 6:131. doi: 10.3389/fnut.2019.00131, 31482093 PMC6710320

[29] Counts BR Buckner SL Mouser JG Dankel SJ Jessee MB Mattocks KT . Muscle growth: to infinity and beyond? Muscle Nerve. (2017) 56:1022–30. doi: 10.1002/mus.25696, 28543604

[30] Barakat C Pearson J Escalante G Campbell B De Souza EO. Body recomposition: can trained individuals build muscle and lose fat at the same time? Strength Cond J. (2020) 42:7–21. doi: 10.1519/ssc.0000000000000584

[31] Kataoka R Hammert WB Yamada Y Song JS Seffrin A Kang A . The plateau in muscle growth with resistance training: an exploration of possible mechanisms. Sports Med. (2024) 54:31–48. doi: 10.1007/s40279-023-01932-y, 37787845

[32] Allerton TD Ravussin E Rood J Irving B Lemoine N Mullenix S . Energy expenditure measured overnight in a whole-room indirect calorimeter in four collegiate American-style football linemen. Med Sci Sports Exerc. (2020) 52:343–3. doi: 10.1249/01.mss.0000677488.79109.1f

[33] Sobolewski EJ Lochbaum M. Estimated energy expenditure during NCAA division I college football practice. J Strength Cond Res. (2019) 31:e1–e245.

[34] Murphy C Koehler K. Energy deficiency impairs resistance training gains in lean mass but not strength: a meta-analysis and meta-regression. Scand J Med Sci Sports. (2022) 32:125–37. doi: 10.1111/sms.14075, 34623696

[35] Halonen EJ Gabriel I Kelahaara MM Ahtiainen JP Hulmi JJ. Does taking a break matter-adaptations in muscle strength and size between continuous and periodic resistance training. Scand J Med Sci Sports. (2024) 34:e14739. doi: 10.1111/sms.14739, 39364857

[36] Cumming KT Reitzner SM Hanslien M Skilnand K Seynnes OR Horwath O . Muscle memory in humans: evidence for myonuclear permanence and long-term transcriptional regulation after strength training. J Physiol. (2024) 602:4171–93. doi: 10.1113/JP285675, 39159314

[37] Gordon AN Blue MN Cabre HE Gould LM Smith-Ryan AE. Body composition of NCAA division I football players pre- and post-COVID-19 stay-at-home advisory. J Sports Med Phys Fitness. (2022) 62:1662–7. doi: 10.23736/S0022-4707.22.13465-1, 35415995

[38] Garthe I Raastad T Refsnes PE Sundgot-Borgen J. Effect of nutritional intervention on body composition and performance in elite athletes. Eur J Sport Sci. (2013) 13:295–303. doi: 10.1080/17461391.2011.643923, 23679146

